# Supplementary material for: C-terminus CD28 phosphorylation (Y218) modulates IL-2 secretion and antitumor effect of CAR-T cells
Source: bioRxiv. 2026 Feb 1:2026.01.28.701378. Preprint. [Version 1] doi: 10.64898/2026.01.28.701378 (PMC12874040; doi:10.64898/2026.01.28.701378)

bioRxiv preprint doi: https://doi.org/10.64898/2026.01.28.701378; this version posted February 1, 2026. The copyright holder for this preprint (which was not certified by peer review) is the author/funder, who has granted bioRxiv a license to display the preprint in perpetuity. It is made available under aCC-BY 4.0 International license.

Supplemetary Figures

Supplementary Figure 1. Expression of PSCA in HPAC^{WT} and HPAC$^{PSCA-KO}$ cells, measured by flow cytometry.

Supplementary Figure 2. A. Expression of CAR, CD4, and CD8 in WT and 218F CAR-T cells 7-10 days after transduction. Each point represents an individual healthy donor (n = 11). Statistical significance was determined by one-way ANOVA. **B.** The lysis of HPAC^{WT} cells when treated with WT or 218F CAR-T cells was evaluated using a real-time cytotoxicity assay (xCELLigence). Left Panel: Representative graph showing % of cytolysis over a 72 hour period after the addition of the effector cells. Right Panel: % of cytolysis of WT and 218F CAR-T cells at 12 hours after the addition of the effector cells. Significance was determined by paired t-test. Each symbol represents an independent experiment using one of five different healthy donors. **C.** Proliferation capacity of WT vs 218F CAR-T cells, analyzed by CellTrace Violet dilution. WT and 218F CAR-T cells were co-cultured at a 2:1 E:T ratio and incubated at 37°C for 4 days. Percentage of Divided cells (left) and percentage of cells in the last division (right) of 218F CAR-T cells is shown normalized to WT CAR-T cells. Significance was determined by t-test with Welch's correction. * = P≤0.05. Each symbol represents an independent experiment from 4 different healthy donors. Data is represented as the mean ± standard deviation (SD). **D.** CD4 and CD8 CAR-T cells were sorted using magnetic MicroBeads. WT and 218F CAR-T cells were cocultured with HPAC^{WT} cells at different CD4:CD8 ratios. Supernatant was collected after 24 hours and cytokine production was measured by ELISA. Left Panel. Representative graph showing the IL-2 production at different CD4:CD8 ratio. Dotted lines indicate IL-2 production by unsorted CAR-T cells. Center and left panel show production of IL-2 production of 218F CAR-T cells with respect to WT CAR-T cells. Significance was determined by t-test with Welch's correction. ** = P≤0.01. Each symbol represents an independent experiment from 3 different healthy donors.

bioRxiv preprint doi: https://doi.org/10.64898/2026.01.28.701378; this version posted February 1, 2026. The copyright holder for this preprint (which was not certified by peer review) is the author/funder, who has granted bioRxiv a license to display the preprint in perpetuity. It is made available under aCC-BY 4.0 International license.

Supplementary Figure 3. Transcriptomic profile of WT vs 218F CAR-T cells. CAR-T cells were cocultured with $HPAC^{WT}$ cells for 24 hours and subsequently sorted into $CD4^+$ and $CD8^+$ subsets using the MACSQuant® Tyto® system prior to RNA extraction. **A.** Principal component analysis (PCA) of WT and 218F CAR-T cells in all samples (left), $CD4^+$ cells (center), and $CD8^+$ cells (right). **B.** Gene expression of genes in the IL-17 signaling pathway. Enriched pathways between WT and 218F CAR-T cells were identified using IPA® software. Bar plots show log_2 fold change in gene expression in 218F CAR-T cells relative to WT CAR-T cells. $CD4^+$ cells are shown on the left and $CD8^+$ cells on the right.

Supplementary Figure 4. A. Expression of ITK in Jurkat cells. ITK-KO Jurkat cells were generated using the CRISPR/Cas9 technology to target exon 1 of ITK. A clonal population was derived by limiting dilution. **B.** CAR transduction efficiency in Jurkat WT and Jurkat ITK-KO 7 days after transduction. Representative plot of one transduction.

bioRxiv preprint doi: https://doi.org/10.64898/2026.01.28.701378; this version posted February 1, 2026. The copyright holder for this preprint (which was not certified by peer review) is the author/funder, who has granted bioRxiv a license to display the preprint in perpetuity. It is made available under aCC-BY 4.0 International license.

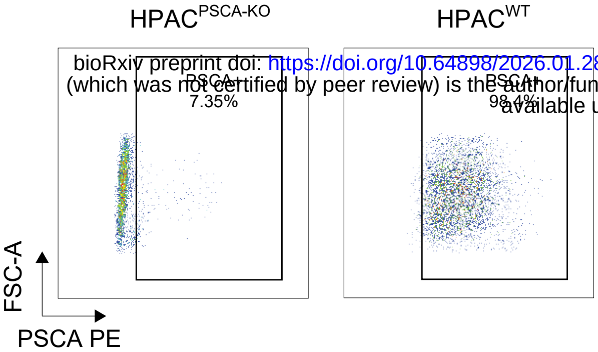

bioRxiv preprint doi: https://doi.org/10.64898/2026.01.28.701378; this version posted February 1, 2026. The copyright holder for t
(which was not certified by peer review) is the author/funder, who has granted bioRxiv a license to display the preprint in perpetuit
available under aCC-BY 4.0 International license.

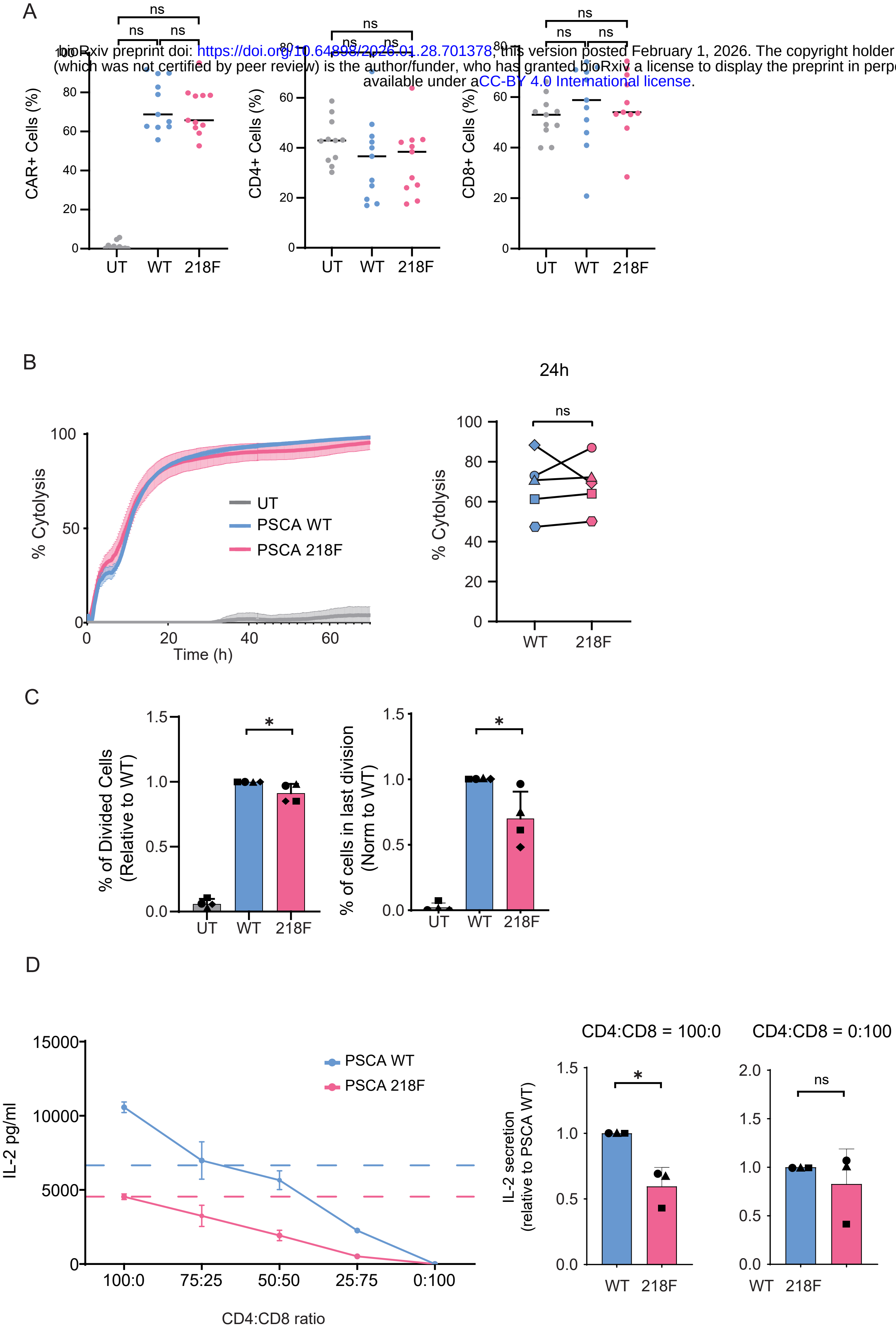

bioRxiv preprint doi: https://doi.org/10.64898/2026.01.28.701378; this version posted February 1, 2026. The copyright holder for t
(which was not certified by peer review) is the author/funder, who has granted bioRxiv a license to display the preprint in perpetuit
available under a CC-BY 4.0 International license.

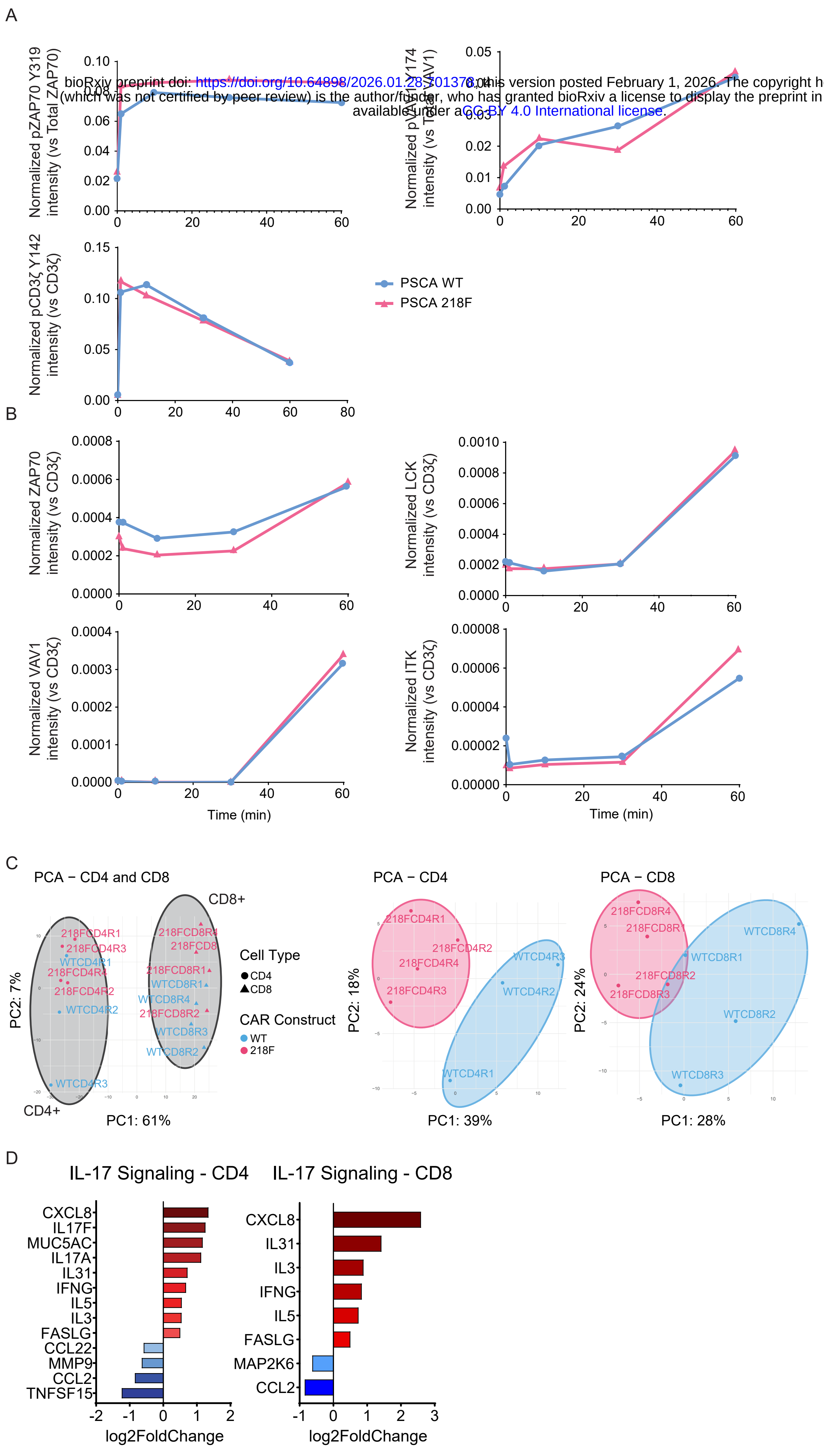

bioRxiv preprint doi: https://doi.org/10.64898/2026.01.28.701378; this version posted February 1, 2026. The copyright holder for t
(which was not certified by peer review) is the author/funder, who has granted bioRxiv a license to display the preprint in perpetuit
available under aCC-BY 4.0 International license.

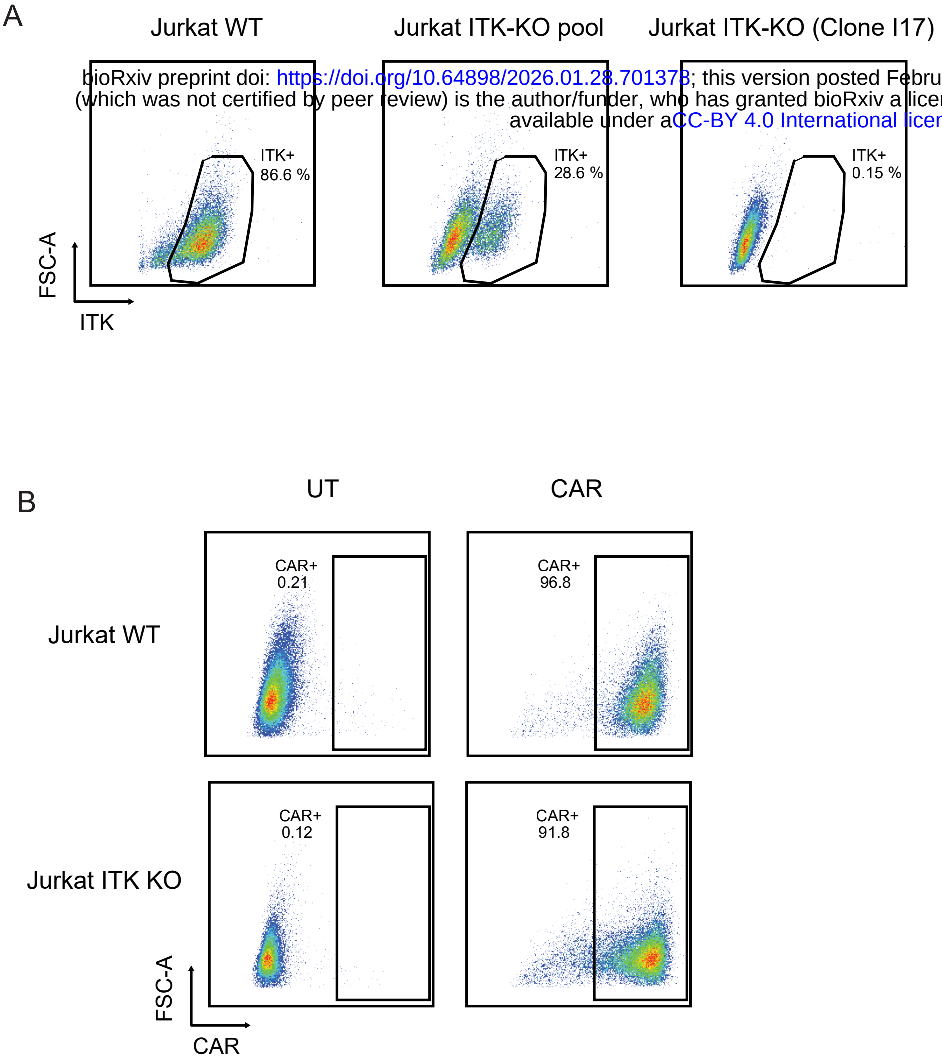

Supplement: 1 [file NIHPP2026.01.28.701378v1-supplement-1.pdf]
